# Efficacy and Safety of CKDB-322, a Combination of *Lactiplantibacillus plantarum* Q180 and *Phaeodactylum tricornutum*, for Reducing Body Fat and Abdominal Adiposity in Overweight Adults

**DOI:** 10.3390/nu18020250

**Published:** 2026-01-13

**Authors:** Hyang-Im Baek, So-Young Kwon, Hye-Ji Noh, Soo Jung Park

**Affiliations:** 1 Department of Food Science & Nutrition, Woosuk University, Wanju 55338, Republic of Korea; hyangim100@gmail.com; 2 Research Institute, Chong Kun Dang Bio (CKD Bio), Seoul 03742, Republic of Korea; ksy2022@ckdbio.com (S.-Y.K.); nhj@ckdbio.com (H.-J.N.); 3 Department of Sasang Constitutional Medicine, College of Korean Medicine, Woosuk University, Jeonju 55338, Republic of Korea

**Keywords:** *Lactiplantibacillus plantarum*, *Phaeodactylum tricornutum*, probiotics, microalgae, anti-obesity, overweight, body fat, abdominal fat, metabolic health

## Abstract

Background: CKDB-322, a combination of *Lactiplantibacillus plantarum* Q180 and *Phaeodactylum tricornutum*, has shown anti-obesity potential in preclinical models, although human evidence is still limited. This randomized, double-blind, placebo-controlled, 12-week trial evaluated the efficacy and safety of CKDB-322 in overweight adults. Methods: Participants were aged 19–65 years; had a body mass index (BMI) of 25–30 kg/m^2^, and a waist circumference of ≥90 cm for men or ≥85 cm for women. They were randomly assigned to receive either CKDB-322, which provided 1.0 × 10^9^ CFU of *L. plantarum* Q180 and 200 mg of *P. tricornutum* daily (*n* = 50), or a placebo (*n* = 50). Results: CKDB-322 supplementation resulted in statistically significant reductions in body fat mass and body fat percentage, as measured by dual-energy X-ray absorptiometry (DEXA), compared to the placebo group (*p* < 0.05). Computed tomography (CT) analyses also revealed significant reductions in abdominal fat area in the CKDB-322 group (*p* < 0.05). Additional improvements were observed in body weight and anthropometric parameters. Among metabolic biomarkers, serum triglycerides and leptin levels decreased significantly in the CKDB-322 group compared to the placebo. Exploratory microbiome analyses indicated an increase in the relative abundance of *Lactobacillus*, suggesting potential modulation of the gut–adipose axis. CKDB-322 was well tolerated, with no clinically significant adverse events or laboratory abnormalities. Conclusions: Collectively, CKDB-322 demonstrated a favorable safety profile and produced statistically significant improvements in multiple adiposity-related outcomes, including reductions in body fat mass, abdominal adiposity, and key anthropometric measures, supporting its potential as a functional ingredient for body fat reduction and metabolic health.

## 1. Introduction

Overweight and obesity, primarily caused by excessive caloric intake and insufficient energy expenditure, have become chronic global health challenges. Their prevalence is rising across all age groups, sexes, and ethnicities, making excess adiposity a significant public health concern worldwide [1,2,3]. Elevated body fat is a central contributor to metabolic dysfunction and is strongly associated with numerous medical complications, including hypertension [4], dyslipidemia [5], diabetes mellitus [6], cardiovascular disease [7], osteoarthritis [8], atherosclerosis [9], and various cancers [10]. Even a modest weight reduction of approximately five to ten percent of initial body weight has been shown to significantly lower cardiometabolic risk. Therefore, it is recommended as a standard therapeutic goal in obesity management [11].

While lifestyle modifications through diet and physical activity are the primary methods for managing overweight, long-term adherence can be challenging for many individuals, often leading to limited or temporary effectiveness [12,13]. More than one hundred twenty pharmacological agents have been investigated for obesity over the past few decades; however, only a small number have received regulatory approval or sustained clinical use. This is largely due to safety concerns, adverse effects such as headache, dizziness, and nausea or vomiting, as well as limited long-term tolerability [14,15,16]. These challenges highlight the need for safer and more accessible alternatives derived from food-based or naturally occurring bioactive compounds.

Probiotics, defined as live microorganisms that confer health benefits when consumed in adequate amounts [17], have garnered considerable attention as a potential strategy for improving metabolic health. Most probiotic strains belong to non-pathogenic, lactic acid-producing bacteria that help maintain gut homeostasis. By producing organic acids, including acetate, probiotics acidify the gut lumen and suppress the growth of harmful bacteria [18]. The gut microbiota plays a pivotal role in energy balance, lipid storage, immune function, and inflammatory regulation [19,20]. Consequently, alterations in microbial composition, short-chain fatty acid profiles, intestinal permeability, and bile acid metabolism are increasingly recognized as key contributors to the pathophysiology of obesity [21,22]. Modulating the gut–adipose axis through targeted probiotic supplementation has emerged as a promising therapeutic approach [23].

*Lactiplantibacillus plantarum* (*L. plantarum*) is a widely studied lactic acid bacterium that is commonly found in fermented foods and plant materials [24,25]. It metabolizes a variety of carbohydrates, producing lactic acid, and is generally regarded as safe [17,26]. Many *L. plantarum* strains have been incorporated into functional foods and dietary supplements because of their strong probiotic properties [27]. These strains have shown beneficial effects on weight regulation, insulin resistance, and lipid profiles, as demonstrated primarily in preclinical studies and supported by emerging human data, largely through modulation of the gut microbiome and host metabolic signaling pathways [28,29,30]. However, clinical evidence remains heterogeneous, and well-controlled trials evaluating standardized formulations with objective adiposity endpoints are still limited. Among them, *L. plantarum* Q180, originally isolated from healthy adult feces, has demonstrated significant reductions in postprandial triglycerides in human studies [31]. Additionally, several studies have reported promising effects on body fat reduction [32,33,34,35].

*Phaeodactylum tricornutum* (*P. tricornutum*) is a microalgal species enriched in eicosapentaenoic acid (EPA)-containing polar lipids and fucoxanthin derivatives [36]. These compounds have been reported to exert antioxidant, anti-inflammatory, and lipid-modulating effects. They have also been investigated for their potential role in regulating body weight and adipose tissue metabolism [37,38,39].

Preclinical investigations [40] of CKDB-322, a combination of *L. plantarum* Q180 and *P. tricornutum*, have indicated complementary metabolic effects that may aid in obesity management. In 3T3-L1 adipocytes, CKDB-322 decreased adipogenesis by downregulating the transcriptional regulators peroxisome proliferator-activated receptor gamma (PPARγ) and CCAAT/enhancer-binding protein alpha (C/EBPα) while also promoting glycerol release. In mice subjected to a high-fat diet, oral administration of CKDB-322 over eight weeks led to reduced body weight gain and adiposity, along with improvements in serum glucose and lipid profiles. These benefits were associated with decreased lipogenic markers and enhanced fatty acid oxidation and energy metabolism pathways, supporting the potential synergistic effects of the combined ingredients.

While preclinical results indicate that CKDB-322 has anti-adipogenic and metabolic regulatory actions through the coordinated modulation of lipid synthesis and fatty acid oxidation pathways, there is still insufficient evidence to confirm these effects in humans. To fill this gap, we designed a randomized, double-blind, placebo-controlled clinical trial to assess the efficacy and safety of CKDB-322 in reducing body fat mass and abdominal adiposity and improving key metabolic parameters in overweight adults.

## 2. Materials and Methods

### 2.1. Ethics

The study was conducted after receiving ethical approval from the Institutional Review Board (IRB) of Woosuk University Korean Medicine Hospital (Protocol Number CKDB-322-01; IRB Number: WSOH IRB H2311-05; approval date: 20 November 2023). The trial was prospectively registered with the Clinical Research Information Service in the Republic of Korea under the identifier KCT0009303. Prior to enrollment, all participants received detailed explanations regarding the study objectives and procedures and voluntarily provided written informed consent. The trial was conducted in compliance with the Declaration of Helsinki, the International Council for Harmonisation Good Clinical Practice guidelines, and the CONSORT recommendations for randomized controlled trials.

### 2.2. Study Participants

This study enrolled 100 adults classified as overweight. According to the World Health Organization (WHO) classification [41], overweight was defined as a body mass index (BMI) of 25.0–29.9 kg/m^2^. Participants were eligible if they met the following inclusion criteria: (1) men or women aged between 19 and 65 years; (2) a BMI between 25 and 30 kg/m^2^, indicating the overweight range; (3) a waist circumference of at least 90 cm for men or 85 cm for women; and (4) voluntary submission of written informed consent prior to participation.

The exclusion criteria for participants were as follows: (1) pregnant or breastfeeding women; (2) women of childbearing potential or men who were unwilling or unable to use reliable contraception throughout the study; (3) women who had given birth within six months prior to screening; (4) individuals with current or past medical conditions, including uncontrolled hypertension, diabetes mellitus, neurological or hormonal disorders affecting body weight, severe psychiatric disorders, alcohol abuse, malignant tumors, significant cerebrovascular or cardiac disease, or any other serious comorbidity deemed clinically relevant by the investigator; (5) individuals who had taken anti-obesity drugs or other medications known to cause significant weight changes; (6) known hypersensitivity to any component of the study product or a history of allergies that the investigator deemed incompatible with study participation; (7) a body weight change of more than 5 percent within one month prior to screening; (8) participation in another clinical study involving investigational drugs, foods, randomized, double-blind, placebo or medical devices within three months before screening; or (9) any other condition that the investigator believed made the participant unsuitable for participation in this clinical trial.

### 2.3. Study Design

A single-center, randomized, double-blind, placebo-controlled clinical trial was conducted at the Korean Medicine Hospitals of Woosuk University (Jeonju, Republic of Korea) over a 12-week intervention period. Recruitment and follow-up assessments took place from January 2024 to September 2024.

**Figure 1:**
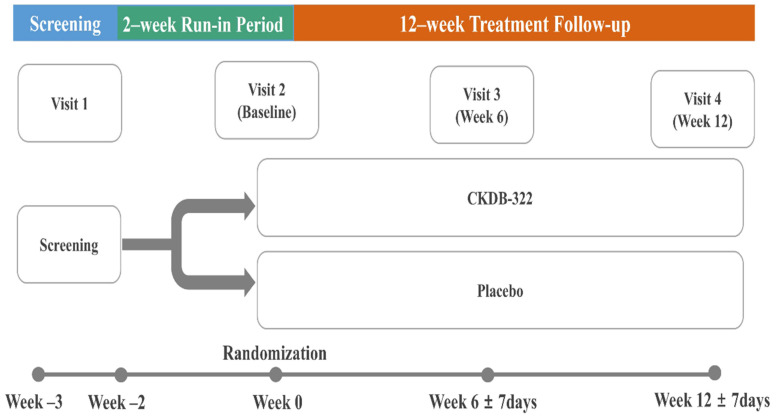
Summary of the study design.

Participants who met the eligibility criteria during the screening visit entered a run-in period. After this phase, investigators conducted a final evaluation to confirm eligibility. Those who continued to meet all criteria were randomly assigned in a 1:1 ratio to receive either CKDB-322 (*n* = 50) or a placebo (*n* = 50). Randomization utilized predefined allocation codes that were securely maintained to ensure blinding for both investigators and participants, with no unblinding events occurring during the trial. Each participant attended four scheduled visits: Visit 1 (week −3), Visit 2 (week 0), Visit 3 (week 6), and Visit 4 (week 12). Baseline assessments were collected during Visit 1 or Visit 2, and outcome measurements were obtained at six-week intervals thereafter. A summary of the study design is shown in Figure 1.

### 2.4. Diet and Physical Activity Counseling

Before randomization, participants underwent a 2-week run-in period where they practiced basic dietary and lifestyle adjustments as instructed by the study staff. During the intervention phase, both groups received standardized guidance on diet and physical activity to minimize differences in lifestyle factors. Participants were advised to reduce their daily energy intake by approximately 500 kcal and to follow a carbohydrate-restricted diet. Additionally, they were encouraged to engage in regular physical activity, aiming for 20 to 30 min of moderate exercise three times per week.

Dietary intake and physical activity were monitored using standardized daily meal record forms and structured physical activity sheets to ensure consistent data collection across participants. Energy and nutrient intakes were evaluated through standardized 3-day dietary records, which included two weekdays and one weekend day. The total daily consumption of energy, carbohydrates, lipids, protein, and dietary fiber was analyzed using a computer-aided nutritional analysis program (CAN-Pro version 6.0, Korean Nutrition Society, Seoul, Republic of Korea). Physical activity levels were assessed using the International Physical Activity Questionnaire (IPAQ), with all responses converted into metabolic equivalent task (MET) values to quantify overall physical activity.

### 2.5. Study Products and Interventions

The study products were supplied by Chong Kun Dang Bio Corporation (Seoul, Republic of Korea). CKDB-322 was a mixed formulation of *L. plantarum* Q180 and *P. tricornutum*, manufactured according to a standardized process outlined in previous studies [40]. In brief, *L. plantarum* Q180 (NCBI genome accession CP073753), originally isolated from the feces of a healthy Korean adult, was cultured in an optimized medium using a controlled bioreactor system, concentrated, and freeze-dried to obtain a stable probiotic powder [32,33,34,35]. *P. tricornutum* is a marine microalga that has been reported in the literature to contain bioactive hydrophobic compounds, including EPA. The fatty acid composition of *P. tricornutum*, including EPA, has been previously investigated and reported in a published preclinical study, in which a total of 37 fatty acids were identified and quantified using gas chromatography–flame ionization detection (GC-FID) [40]. The *P. tricornutum* biomass was cultivated in a controlled photobioreactor system under standardized environmental and nutrient conditions. After reaching the optimal growth phase, the culture was harvested by centrifugation, heat-treated, and freeze-dried to produce a stable lyophilized microalgal powder [38,39]. The two powders were then uniformly blended in predefined ratios without further modification to generate the final active CKDB-322 formulation.

For the clinical trial, CKDB-322 was formulated as a hard capsule. Participants took one capsule twice daily for 12 weeks, resulting in a total daily intake of 1.0 × 10^9^ CFU (100 mg) of *L. plantarum* Q180 and 200 mg of *P. tricornutum*. Microcrystalline cellulose and magnesium stearate served as excipients, while the placebo contained only these excipients without any active ingredients. All study products were manufactured in Good Manufacturing Practice (GMP)-certified facilities and were indistinguishable in appearance, weight, and packaging to ensure blinding. Compliance was monitored through capsule counts and participant logs at each study visit.

### 2.6. Efficacy Outcome Measures

The primary efficacy outcome was the change in body fat mass from baseline to week 12, assessed by dual-energy X-ray absorptiometry (DEXA; Primus, OsteoSys, Seoul, Republic of Korea). The DEXA system was calibrated regularly according to the manufacturer’s instructions, and all scans were performed by trained technicians using standardized procedures. Participants were scanned in the supine position while wearing light clothing and no metal accessories.

Secondary efficacy outcomes included additional body composition, anthropometric, and metabolic parameters. Body fat percentage, lean body mass, and regional body composition were also evaluated using the same DEXA system. Abdominal adiposity was assessed by computed tomography (CT; Bright Speed Elite Select, GE, Beijing, China), measuring total abdominal fat area, visceral fat area, and subcutaneous fat area. Anthropometric variables, including BMI, body weight, waist circumference, hip circumference, and waist-to-hip ratio (WHR), were measured using standardized procedures and identical instruments.

Fasting venous blood samples were collected after an overnight fast of at least 8 h at baseline and week 12. Blood samples were obtained from the antecubital vein, and serum or plasma was separated by centrifugation. Samples were either analyzed immediately for routine biochemical parameters or aliquoted and stored at −80 °C until further analysis. Serum lipid profiles, including total cholesterol, triglycerides, high-density lipoprotein cholesterol (HDL-C), and low-density lipoprotein cholesterol (LDL-C), were obtained using a standardized automated biochemical analyzer (AU480, Beckman Coulter, Tokyo, Japan). Serum adipokines and cytokines were measured from fasting blood samples. Adiponectin and leptin concentrations were quantified using ELISA kits from Millipore (Billerica, MA, USA), while levels of tumor necrosis factor-alpha (TNF-α), interleukin-1 beta (IL-1β), and interleukin-6 (IL-6) were determined using ELISA kits from R&D Systems (Minneapolis, MN, USA), following the manufacturers’ protocols.

All measurements were performed using the same equipment at each visit. Whenever possible, assessments were performed by the same trained examiner to minimize inter-operator variability.

### 2.7. Microbiome and Short-Chain Fatty Acid Analysis

#### 2.7.1. Fecal Sample Collection

Fecal samples were collected at baseline and at the end of the 12-week intervention. Participants were provided with a standardized stool collection kit (mediclin^®^ Stool Container, DM.Con 3040; DAIHAN Medical, Seoul, Republic of Korea) and instructed to collect samples at home following written guidelines. The collected samples were promptly stored at −80 °C until further analysis.

#### 2.7.2. Microbiome Analysis

Genomic DNA was isolated from fecal samples, and the V3–V4 region of the 16S rRNA gene was amplified using primers 341F and 805R. Library construction and sequencing were performed by the National Instrumentation Center for Environmental Management (NICEM, Seoul, Republic of Korea) using the Illumina MiSeq platform(Illumina Inc., San Diego, CA, USA; https://www.illumina.com/systems/sequencing-platforms/miseq.html (accessed on 10 January 2026)).

Raw reads were processed with QIIME2, and denoising was performed using DADA2 to generate amplicon sequence variants (ASVs). Taxonomic assignments were made using a validated reference database. Differential abundance analyses were conducted at the family and genus levels, focusing on taxa such as *Lactobacillaceae*, *Bifidobacteriaceae*, *Lactobacillus*, and *Bifidobacterium*. Group differences in relative abundance were evaluated using the Wilcoxon rank-sum test. Participants with incomplete fecal sampling were excluded, resulting in 82 participants included in the microbiome dataset (CKDB-322: 43; placebo: 39).

#### 2.7.3. Short-Chain Fatty Acid Analysis

Short-chain fatty acids (SCFAs) in fecal samples were quantified, specifically acetic acid, propionic acid, and butyric acid, which are the primary fermentation-derived fatty acids produced by gut microbiota. SCFA measurements were conducted using gas chromatography–mass spectrometry (GC–MS; Hewlett Packard Model 7890 equipped with a DB-FATWAX Ultra Inert column) at the Korea Research Institute of Biomedical Science (KRIBS, Daejeon, Republic of Korea). Sample preparation and analytical procedures adhered to standard validated protocols. A total of 60 participants provided suitable paired fecal samples for SCFA analysis (CKDB-322: 32; placebo: 28).

### 2.8. Safety Outcome Measures

Safety was assessed throughout the study using various clinical and laboratory parameters. Adverse events reported by participants or observed by investigators were documented at each visit, including onset, duration, severity, and the investigator’s assessment of causality. Vital signs, including systolic blood pressure (SBP), diastolic blood pressure (DBP), pulse rate, and body temperature, were measured at every scheduled visit under standardized resting conditions.

Safety laboratory evaluations were conducted using fasting blood and urine samples, performed at baseline and at the end of the intervention period. These assessments included hematology, serum biochemistry, and urinalysis. Hematology analyses measured red blood cell (RBC) and white blood cell (WBC) counts with differential (neutrophils, lymphocytes, monocytes, eosinophils, and basophils), hemoglobin, hematocrit, and platelet counts. Serum biochemical analyses included measurements of albumin, total protein, glucose, blood urea nitrogen (BUN), serum creatinine, uric acid, total bilirubin, aspartate aminotransferase (AST), alanine aminotransferase (ALT), alkaline phosphatase (ALP), phosphorus, calcium, sodium, potassium, and chloride. Urinalysis assessed specific gravity, pH, protein (albumin), glucose, bilirubin, ketones, WBC, and blood (RBC). All laboratory tests were conducted using consistent analytic platforms across visits to ensure the reliability and comparability of safety data.

### 2.9. Statistical Analysis

The primary objective of this clinical trial was to evaluate the efficacy of CKDB-322 in reducing body fat mass compared to a placebo. The sample size was determined based on the statistical hypothesis regarding the between-group difference in the change in body fat mass from baseline. This estimation was informed by a previous randomized clinical study with similar endpoints and study design [42]. With an equal allocation to the test and placebo groups (1:1 ratio), a two-sided significance level (α) of 0.05, and a statistical power (1 − β) of 90%, the minimum number of evaluable participants was determined to be 40 per group, totaling 80 participants. To account for an anticipated dropout rate of approximately 20%, the final target sample size was set at 100 participants.

Efficacy analyses were primarily conducted using the full analysis set (FAS), with additional supportive analyses carried out in the per-protocol set (PPS). Safety assessments were based on the safety set (SS), which included all participants who received at least one dose of the study product. Continuous variables were summarized as means ± standard deviations (SD), while categorical variables were presented using frequencies and percentages.

Within-group changes from baseline were assessed using Paired *t*-test when normality assumptions were met and Wilcoxon signed-rank test when the data were non-normally distributed. Between-group comparisons were performed using Independent *t*-test or Wilcoxon rank-sum test, based on the distribution characteristics. For categorical variables, differences between-group were evaluated using Chi-square test or Fisher’s exact test if the expected cell counts were inadequate.

All statistical analyses were conducted using SAS^®^ software (Version 9.4, SAS Institute, Cary, NC, USA). Two-sided tests were applied, with a *p*-value of less than 0.05 considered statistically significant.

## 3. Results

### 3.1. Participant Characteristics

**Figure 2:**
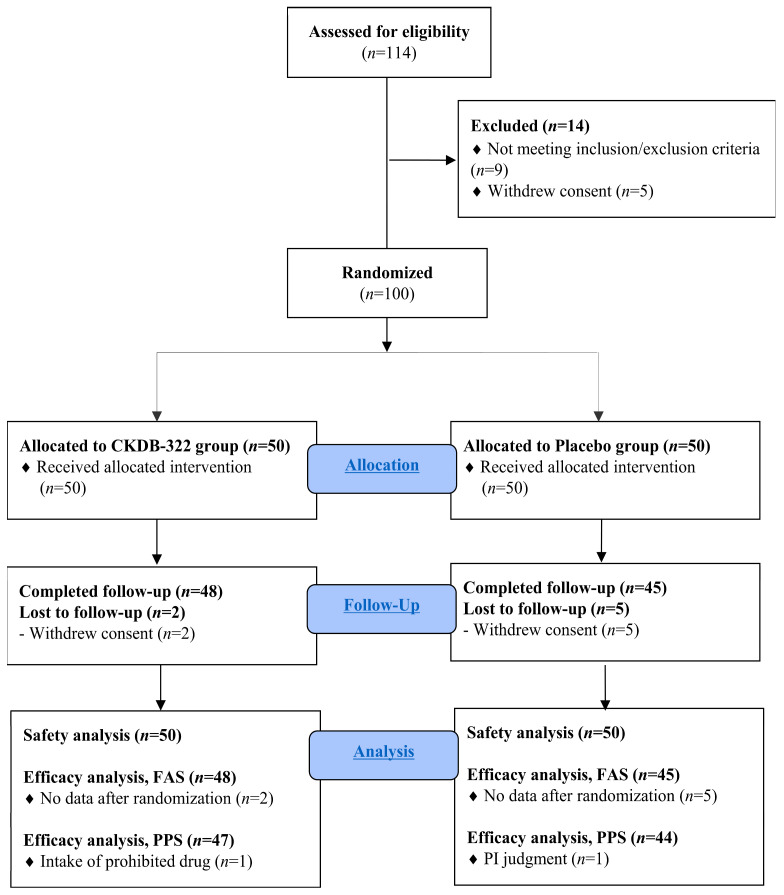
The CONSORT flow diagram of the study.

A total of 114 volunteers were screened for eligibility before enrollment. As illustrated in the CONSORT flow diagram (Figure 2), 14 participants did not proceed to randomization. Nine individuals were excluded for not meeting the predefined inclusion or exclusion criteria, while five withdrew consent during the screening process. Consequently, 100 participants met all eligibility requirements and were randomly assigned to either the CKDB-322 group (*n* = 50) or the placebo group (*n* = 50). During the 12-week intervention, seven participants discontinued the study before completion. Two participants in the CKDB-322 group and five in the placebo group withdrew consent during the intervention period. Importantly, no participants dropped out due to insufficient compliance, reflecting a high level of adherence throughout the trial. Ultimately, 93 participants completed all study visits.

The FAS included 93 participants (CKDB-322: *n* = 48; placebo: *n* = 45), excluding the seven individuals who withdrew before the primary efficacy outcome was assessed. The PPS comprised 91 participants (CKDB-322: *n* = 47; placebo: *n* = 44). One participant in the CKDB-322 group was excluded from the PPS due to the use of a prohibited concomitant medication, and one participant in the placebo group was excluded following the investigator’s judgment that vestibular neuronitis and subsequent prolonged gastrointestinal symptoms could have compromised the reliability of efficacy assessments.

Table 1 presents the baseline demographic and clinical characteristics of the participants. Baseline demographic and clinical characteristics, including sex distribution, age, anthropometric measures, and body fat mass, did not differ significantly between the CKDB-322 and placebo groups (all *p* > 0.05). The mean age of participants in both groups was approximately 36 years, with a higher proportion of female participants in each group. The anthropometric measures, including BMI and waist circumference, were similar between the CKDB-322 and placebo groups, indicating successful randomization and balanced baseline profiles. These findings confirm that the treatment groups were equivalent at baseline, allowing for an unbiased evaluation of the intervention effects.

### 3.2. Dietary Intake and Physical Activity

Dietary intake and physical activity were assessed to ensure that lifestyle patterns remained similar between the two groups throughout the intervention (Table 2). Both groups received standardized counseling on caloric reduction and moderate exercise, resulting in modest changes in energy and macronutrient intakes that did not differ significantly between them. Over the 12-week period, total energy intake slightly decreased in both groups, with similar trends noted in carbohydrate, lipid, protein, and fiber consumption.

Physical activity levels, measured using the IPAQ and expressed as MET-min/week, increased for both groups during the intervention. While the CKDB-322 group showed a greater numerical increase, the difference between the groups was not statistically significant.

Overall, these findings suggest that dietary intake and physical activity patterns were generally comparable between the two groups, indicating that lifestyle-related factors are unlikely to explain the differences observed in body fat outcomes.

### 3.3. Efficacy Evaluation

Efficacy outcomes were evaluated using the FAS and measured before the intervention and at 12 weeks after. PPS analyses were also conducted to support the robustness of the findings, presented separately in Supplementary Table S1. Overall, both within-group and between-group comparisons consistently demonstrated the efficacy of CKDB-322 over 12 weeks.

**Figure 3:**
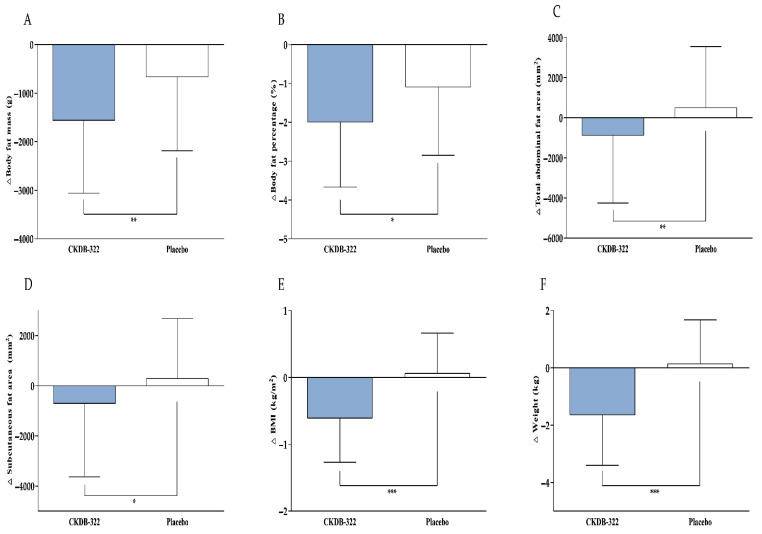

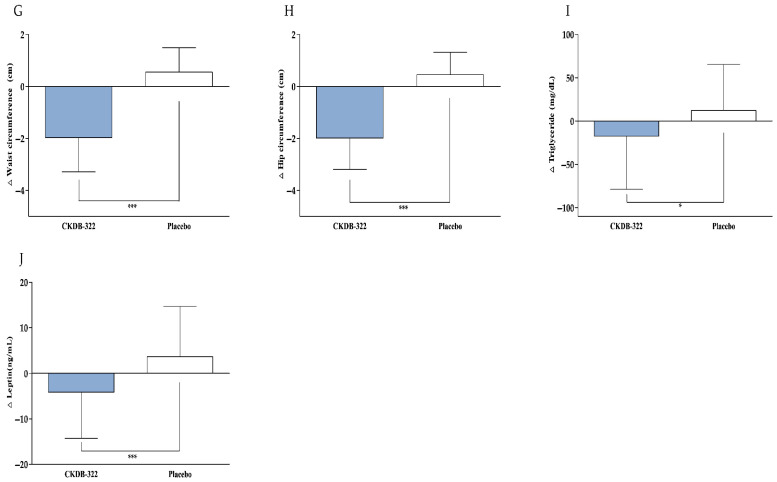
Changes in efficacy outcomes after 12-week intervention. (**A**) Body fat mass, (**B**) body fat percentage, (**C**) total abdominal fat area, (**D**) subcutaneous fat area, (**E**) BMI, (**F**) weight, (**G**) waist circumference, and (**H**) hip circumference, (**I**) triglycerides, and (**J**) leptin in the CKDB-322 and placebo groups at baseline and after 12 weeks of intervention. Values are presented as mean ± SD. Analyzed by Wilcoxon rank-sum test or Independent *t*-test for change value between the groups. * *p* < 0.05, ** *p* < 0.01, *** *p* < 0.001 vs. placebo group.

The efficacy outcomes for body composition, abdominal fat distribution, and anthropometric parameters are summarized in Table 3 and illustrated in Figure 3. The primary efficacy endpoint, body fat mass, significantly decreased after 12 weeks of CKDB-322 supplementation compared to baseline (mean change: −1559.19 ± 1500.58 g, *p* < 0.0001). The placebo group also showed a significant within-group reduction (−663.78 ± 1523.66 g, *p* = 0.0055), but the magnitude of change was significantly greater in the CKDB-322 group (*p* = 0.0053).

Body fat percentage also declined in both groups (CKDB-322: −1.99 ± 1.68%, *p* < 0.0001; placebo: −1.09 ± 1.76%, *p* = 0.0001), with a significantly larger reduction in the CKDB-322 group (*p* = 0.0452). In contrast, the 12-week change in lean body mass did not differ significantly between groups (CKDB-322: 545.90 ± 774.16 g; placebo: 779.87 ± 1051.00 g; *p* > 0.05), indicating that fat reduction occurred without a loss of lean tissue.

**Figure 4:**
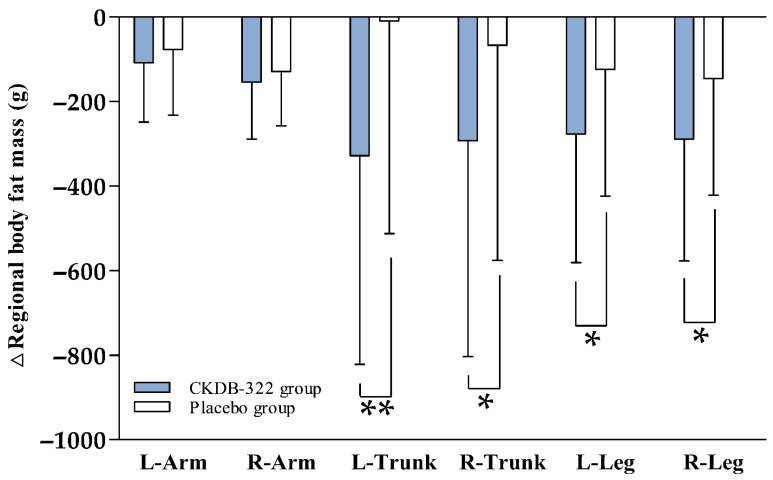
Changes in regional body fat mass after 12-week intervention. Anatomical region (left arm, right arm, left trunk, right trunk, left leg, and right leg) in the CKDB-322 and placebo groups at baseline and after 12 weeks of intervention. Values are presented as mean ± SD. Analyzed by Independent *t*-test for change value between the groups. * *p* < 0.05, ** *p* < 0.01 vs. placebo group.

In addition to whole-body indices, regional fat distribution was evaluated to further characterize the fat-loss patterns associated with CKDB-322 supplementation. Overall, reductions were consistently greater in the CKDB-322 group than in the placebo group (Figure 4). In the upper arms, decreases were modest for both groups; however, the CKDB-322 group showed slightly larger reductions in the left arm (−108.54 ± 140.35 g vs. −77.29 ± 155.06 g) and the right arm (−153.88 ± 134.82 g vs. −129.00 ± 128.51 g), although these between-group differences were not statistically significant (*p* > 0.05). In contrast, more pronounced effects were observed in the trunk region. The CKDB-322 group exhibited substantial decreases in left trunk fat mass (−328.19 ± 493.39 g), while the placebo group showed virtually no change (−9.49 ± 503.04 g), resulting in a significant between-group difference (*p* = 0.0027). A similar pattern was observed in the right trunk, with reductions of −292.60 ± 510.68 g in the CKDB-322 group compared to −66.87 ± 508.97 g in the placebo group (*p* = 0.0356). These findings indicate that CKDB-322 had a measurable impact on central fat accumulation, an important metabolic depot. Reductions were also observed in the lower legs. The CKDB-322 group exhibited significantly greater decreases in both left leg fat mass (−276.50 ± 304.79 g) and right leg fat mass (−288.79 ± 288.59 g) compared to the placebo group (−124.11 ± 299.47 g and −145.89 ± 275.70 g, respectively). Between-group comparisons confirmed significant differences for both legs (*p* = 0.0171 and *p* = 0.0167). Overall, these findings indicate that CKDB-322 supplementation resulted in greater reductions in regional body fat mass than placebo, with particularly pronounced effects in the trunk and lower extremities.

Abdominal fat parameters assessed by CT showed favorable changes with CKDB-322 supplementation. The total abdominal fat area significantly decreased in the CKDB-322 group (−872.98 ± 3375.92 mm^2^, *p* = 0.0157), while the placebo group showed no significant change (498.16 ± 3047.58 mm^2^, *p* = 0.2788). The between-group difference was significant (*p* = 0.0086). Subcutaneous fat area also decreased significantly in the CKDB-322 group (−705.06 ± 2932.64 mm^2^, *p* = 0.0028), but did not change significantly in the placebo group (290.84 ± 2387.70 mm^2^, *p* = 0.2964), resulting in a significant between-group difference (*p* = 0.0196). Visceral fat area slightly decreased in the CKDB-322 group (−167.92 ± 1440.81 mm^2^, *p* = 0.1251) and increased in the placebo group (207.31 ± 1380.54 mm^2^, *p* = 0.3193), indicating a trend favoring CKDB-322 in the between-group comparison (*p* > 0.05).

Anthropometric outcomes, including body weight, BMI, waist circumference, hip circumference, and WHR, demonstrated favorable changes with CKDB-322 supplementation. After 12 weeks, the CKDB-322 group showed significant reductions in body weight (−1.64 ± 1.76 kg, *p* < 0.0001), while the placebo group showed no significant change (0.14 ± 1.53 kg, *p* = 0.5426). The between-group differences were significant (*p* < 0.0001). BMI decreased significantly in the CKDB-322 group (−0.61 ± 0.66 kg/m^2^, *p* < 0.0001), whereas no meaningful change was observed in the placebo group (0.06 ± 0.60 kg/m^2^, *p* = 0.5067), resulting in a significant between-group difference (*p* < 0.0001). Waist circumference decreased markedly in the CKDB-322 group (−1.97 ± 1.32 cm, *p* < 0.0001) but increased significantly in the placebo group (0.55 ± 0.94 cm, *p* = 0.0003), yielding a strong between-group difference (*p* < 0.0001). Hip circumference also decreased significantly in the CKDB-322 group (−1.99 ± 1.20 cm, *p* < 0.0001), while it increased in the placebo group (0.45 ± 0.86 cm, *p* = 0.0011), resulting in a significant between-group difference (*p* < 0.0001). WHR showed small but statistically significant changes in both groups, with a greater reduction observed in the CKDB-322 group (*p* = 0.0004). Overall, 12 weeks of CKDB-322 supplementation led to reductions in whole-body and abdominal adiposity, as well as improvements in anthropometric parameters, with several measures showing significantly greater decreases in the CKDB-322 group compared to the placebo group.

Changes in serum lipid profiles, adipokines, and cytokines are summarized in Table 4 and Figure 3. In the CKDB-322 group, triglyceride levels showed a favorable trend, decreasing by −17.48 ± 61.32 mg/dL, while the placebo group experienced an increase of 12.33 ± 53.06 mg/dL. The comparison between groups revealed a statistically significant difference (*p* = 0.0211), indicating that CKDB-322 supplementation helped mitigate the triglyceride increase seen in the placebo group. Additionally, HDL-cholesterol increased significantly in the CKDB-322 group (2.23 ± 7.28 mg/dL, *p* = 0.0063), although the difference between groups was not statistically significant (*p* > 0.05). Total cholesterol and LDL-cholesterol levels remained stable over the 12-week period, showing no significant changes within either group or between groups. Regarding adipokines, serum leptin levels decreased significantly in the CKDB-322 group (−4.14 ± 10.18 ng/mL, *p* = 0.0071), while the placebo group saw an increase of 3.65 ± 11.02 ng/mL (*p* = 0.0317). The between-group comparison confirmed a highly significant difference (*p* = 0.0006), suggesting a notable effect of CKDB-322 on leptin regulation. No significant differences were observed in adiponectin, TNF-α, IL-1β, and IL-6 between the CKDB-322 and placebo groups after 12 weeks of intervention. Overall, CKDB-322 supplementation was associated with significant reductions in serum triglycerides and leptin levels, while other metabolic markers showed no significant differences between groups.

### 3.4. Results of Microbiome and Short-Chain Fatty Acid Analysis

**Figure 5:**
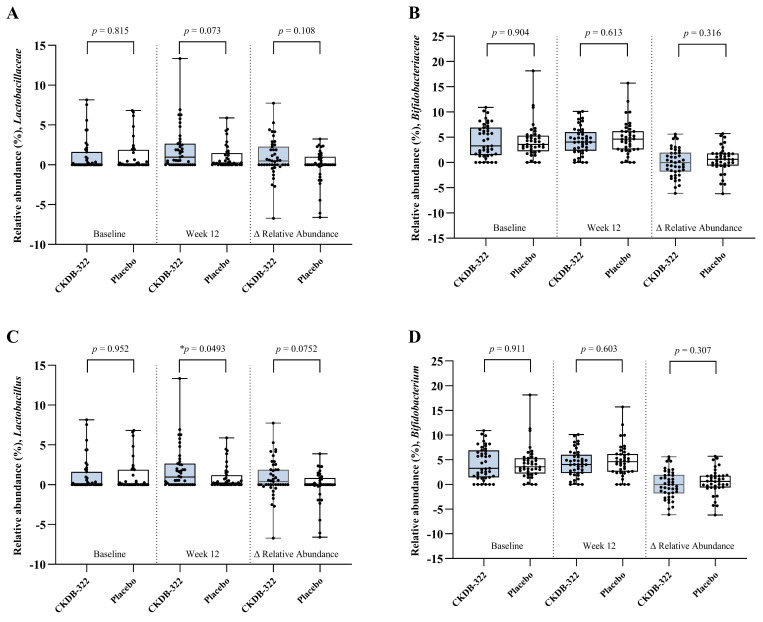
Relative abundance of gut microbial taxa. Taxonomic profiling of fecal samples collected at baseline and week 12 was conducted to evaluate the relative abundance of specific gut microbial taxa in the CKDB-322 and placebo groups. Panels show results for (**A**) *Lactobacillaceae* and (**B**) *Bifidobacteriaceae* at the family level, and (**C**) *Lactobacillus* and (**D**) *Bifidobacterium* at the genus level. Each panel displays group comparisons at baseline (**left**), week 12 (**middle**), and the change from baseline to week 12 (Δ; (**right**)). Values are presented as mean ± SD. Between-group comparisons at each time point and for Δ relative abundance (Week 12 − Baseline) were performed using Wilcoxon rank-sum test. * *p* < 0.05.

Table 5 and Figure 5 summarize the changes in gut microbiota composition and fecal SCFAs following the 12-week intervention. Differential abundance analysis was conducted at the family and genus levels to determine whether CKDB-322 supplementation influenced specific microbial taxa linked to metabolic health. At the family level, no statistically significant differences were found between-group for *Lactobacillaceae* or *Bifidobacteriaceae* after the intervention (*p* > 0.05). Similarly, at the genus level, *Bifidobacterium* did not exhibit significant within-group or between-group differences. In contrast, the relative abundance of *Lactobacillus* (including *Lactiplantibacillus*) was significantly higher in the CKDB-322 group compared to the placebo group at week 12 (*p* = 0.0493). However, the increase in *Lactobacillus* from baseline (Δ value) did not achieve statistical significance between groups (*p* = 0.0752), despite a similar increasing trend.

Fecal SCFA concentrations, including acetic, propionic, and butyric acid, were also analyzed. As shown in Table 5, acetic acid levels rose significantly from baseline in both the CKDB-322 group (13.44 ± 37.32 µmol/g, *p* = 0.0433) and the placebo group (28.69 ± 28.85 µmol/g, *p* < 0.0001). However, the difference in change values between the groups did not reach statistical significance (*p* > 0.05). Propionic acid showed only a minimal and nonsignificant change in the CKDB-322 group (0.38 ± 33.57 µmol/g, *p* = 0.6117), while the placebo group demonstrated a significant increase (13.00 ± 25.13 µmol/g, *p* = 0.0048). Nevertheless, the comparison between groups remained nonsignificant (*p* = 0.0765). Butyric acid levels increased modestly in both groups, with no significant within-group or between-group differences (all *p* > 0.05).

### 3.5. Safety Evaluation

Safety was assessed in all participants within the safety set. During the 12-week intervention, one adverse event occurred in the CKDB-322 group, while two cases were reported in the placebo group. Specifically, the CKDB-322 group had one case of herpes zoster, and the placebo group reported one case each of vestibular neuronitis and dysmenorrhea. There were no statistically significant differences in the incidence of adverse events between the two groups (*p* > 0.05), and all events were determined to be unrelated to the study product. No serious adverse events were noted during the study.

Additionally, vital signs were monitored throughout the study. Measurements of SBP, DBP, pulse rate, and body temperature showed no significant differences between the CKDB-322 and placebo groups (all *p* > 0.05).

Clinical laboratory assessments, including hematology, serum biochemistry, and urinalysis, did not reveal any clinically meaningful abnormalities in either group at any time point.

## 4. Discussion

This study presents the first clinical evaluation of CKDB-322, a combination of *L. plantarum* Q180 and *P. tricornutum*. Over a 12-week intervention, CKDB-322 supplementation resulted in significant improvements in various adiposity-related outcomes. Participants experienced reductions in body fat mass and percentage, as well as decreases in abdominal fat indices (both total and subcutaneous), and key anthropometric measures, including body weight, BMI, waist circumference, hip circumference, and waist-to-hip ratio (WHR). Additionally, serum triglycerides and leptin levels decreased significantly compared to the placebo group. Exploratory microbiome profiling revealed a selective increase in *Lactobacillus*, and CKDB-322 was well tolerated, with no product-related adverse events reported. Taken together, the preferential reduction in adipose tissue without compromise of lean mass supports the biological relevance of CKDB-322 as a safe and potentially effective body-composition-modulating intervention in overweight adults.

CKDB-322 combines two bioactive components that have been previously shown to influence lipid metabolism and adiposity in preclinical studies. *L. plantarum* Q180 has demonstrated anti-obesity effects across multiple experimental models by inhibiting adipocyte differentiation, reducing lipid droplet formation in 3T3-L1 cells, decreasing adipocyte size in high-fat diet (HFD)-fed mice, and improving circulating lipid parameters [32,33,34,35]. The current clinical findings build upon existing preclinical evidence by demonstrating a broader efficacy, particularly regarding abdominal adiposity, a type of fat closely associated with cardiometabolic disorders. Unlike previous studies that primarily examined body weight or adipocyte morphology, CKDB-322 has been shown to significantly reduce abdominal fat area, which has important clinical implications. This indicates that combining *L. plantarum* Q180 with *P. tricornutum* may yield a more comprehensive anti-adiposity effect than using *L. plantarum* Q180 alone.

*P. tricornutum* is a unicellular marine diatom known for its rich nutritional profile, which includes long-chain omega-3 fatty acids like EPA, high-quality protein, dietary fiber, essential minerals, and various carotenoid pigments such as fucoxanthin, which has been extensively studied for its anti-obesity properties [37,38,39,43,44,45,46,47]. Fucoxanthin mediates anti-obesity effects through several mechanisms, including reduced lipid accumulation, inhibition of lipogenic gene expression, enhanced fatty acid oxidation, and the promotion of uncoupling protein-1 (UCP-1) mediated thermogenesis. Recent evidence from a randomized clinical trial of a standardized *P. tricornutum* extract showed beneficial effects on metabolic health indicators, including bone-related parameters and serum lipid profiles [48]. The present study builds on these findings by demonstrating that CKDB-322, a combination of *P. tricornutum* and *L. plantarum* Q180, led to significant reductions in abdominal fat area, subcutaneous fat area, and various anthropometric parameters. This suggests that CKDB-322 may have broader effects than either component alone.

Body composition was measured using DEXA, a highly precise method for quantifying fat, lean, and bone compartments [49]. Due to its sensitivity, DEXA is widely used in clinical trials that assess adiposity-related outcomes [50]. In the present study, 12 weeks of CKDB-322 supplementation resulted in significant reductions in both body fat mass and body fat percentage. These findings align with clinical trials investigating other *L. plantarum* strains, such as LMT1-48 [51] and SKO-001 [52], which also showed notable improvements in adiposity parameters compared to placebo. This supports the reproducibility of body-fat reduction effects across different strains of this species. The observed difference in body fat mass reduction between groups in our trial (−0.90 kg; 95% CI −1.52 to −0.27) is consistent with the pooled estimate from a comprehensive meta-analysis of probiotic interventions (−0.96 kg; 95% CI −1.21 to −0.71) [53]. The observed reduction in fat mass is biologically relevant, particularly in the context of a relatively short intervention period and standardized lifestyle conditions. Previous studies have shown that even moderate reductions in adiposity are associated with improvements in metabolic risk markers and adipose tissue function in overweight individuals [54].

Reductions in trunk fat mass were more significant than those in extremity fat, which aligns with evidence that central adipose depots respond more effectively to weight-loss interventions [55,56]. Because central adiposity carries a disproportionately higher metabolic risk, these findings highlight the clinical significance of CKDB-322-induced fat loss. Importantly, both groups experienced modest increases in lean body mass, indicating that CKDB-322-induced fat reduction occurred without concomitant loss of lean tissue. Preservation of lean mass during fat or weight reduction is clinically important, as loss of fat-free mass is associated with reductions in basal metabolic rate, impaired physical function, and increased risk of weight regain [57,58]. The maintenance of lean mass observed in this study therefore represents a favorable characteristic of CKDB-322, supporting its potential utility for sustainable body-composition improvement.

CT imaging further confirmed the adiposity-lowering effects of CKDB-322. CT is known for its high accuracy in quantifying regional adipose depots, as it can clearly differentiate between subcutaneous and visceral fat using precise tissue density measurements [59,60]. In our study, 12 weeks of CKDB-322 supplementation significantly reduced both total and subcutaneous abdominal fat areas. While reductions in visceral fat area did not reach statistical significance, the improvements in subcutaneous fat, which represents a major component of abdominal adiposity [61], are clinically meaningful and consistent with DEXA findings. These multimodal assessments suggest that CKDB-322 may beneficially modulate abdominal fat distribution and overall body adiposity.

CKDB-322 supplementation also resulted in favorable changes in anthropometric measures. Reductions in body weight and BMI (−1.78 kg and −0.67 kg/m^2^ compared to placebo) were comparable to, and in some cases exceeded, those reported in meta-analyses of probiotic interventions (approximately −0.26 to −0.94 kg for body weight and −0.55 to −0.73 kg/m^2^ for BMI) [53,62].

Additionally, CKDB-322 produced significant improvements in central obesity-related anthropometric measures, including waist circumference, hip circumference, and WHR. These indices are widely recognized as surrogates for visceral fat accumulation and have stronger associations with cardiometabolic risk than body weight or BMI alone [63,64]. Notably, the reduction in waist circumference observed in this trial (−2.52 cm vs. placebo) exceeded the effect sizes typically reported in probiotic meta-analyses, which documented more modest decreases of −1.31 cm [53] and −0.71 cm [62]. This significant reduction highlights CKDB-322’s strong effect on central adiposity, and the accompanying improvement in waist-to-hip ratio (WHR) indicates a shift toward a healthier fat distribution. Together, these findings suggest that CKDB-322 may positively influence visceral fat-related measurements, which are important predictors of cardiometabolic disease risk.

In addition to reductions in adiposity-related outcomes, CKDB-322 also led to significant improvements in metabolic biomarkers. The CKDB-322 group experienced a notable reduction in serum triglycerides compared to the placebo group. Preclinical studies [40] provide mechanistic evidence that supports this finding. In a mouse model on a high-fat diet, serum triglyceride levels rose sharply compared to those on a normal diet; however, CKDB-322 administration significantly mitigated this increase. In contrast, neither *L. plantarum* Q180 nor *P. tricornutum* administered individually produced similar effects, indicating that the combined formulation offers broader metabolic benefits than either component alone. Overall, these preclinical results are consistent with the triglyceride-lowering effect observed in the current trial and suggest a complementary mechanism of action for the mixed preparation. Serum leptin levels significantly decreased in the CKDB-322 group, while they increased in the placebo group. Leptin, an adipocyte-derived hormone, is closely associated with body fat mass and adipocyte hypertrophy. Elevated circulating leptin levels are a hallmark of leptin resistance, commonly observed in individuals with obesity [65,66]. High leptin levels indicate impaired hypothalamic responsiveness, leading to increased appetite and reduced energy expenditure despite excess body fat [67]. The reduction in leptin observed in this trial may indicate improved adipose tissue function and potentially enhanced leptin sensitivity. Given that human probiotic trials have reported inconsistent effects on leptin, the clear and opposing changes between groups underscore a significant metabolic effect of CKDB-322. Together, the concurrent reductions in triglycerides and leptin reinforce the broader metabolic benefits of CKDB-322 and support its potential as a functional intervention for modulating both adiposity and obesity-related endocrine–metabolic dysregulation.

Microbiome analysis supports the observed clinical outcomes, revealing that the CKDB-322 group had a significantly higher relative abundance of the genus *Lactobacillus*, including recently reclassified taxa like *Lactiplantibacillus*, at 12 weeks. However, the differences in changes between groups did not reach statistical significance. This selective modulation of taxonomy, occurring without broader alterations in the overall microbial community structure, suggests that *L. plantarum* Q180 either successfully colonized the gut or promoted the proliferation of native *Lactobacillus* species. Previous preclinical studies have shown that *L. plantarum* Q180 has direct anti-obesity effects, such as suppressing adipocyte differentiation and improving serum lipid profiles [32,34,35]. This increase in *Lactobacillus* abundance may contribute to the observed metabolic benefits, potentially through mechanisms that enhance gut barrier integrity, regulation of SCFAs, and modulation of gut–adipose signaling pathways.

SCFA analysis further supported a beneficial gut-metabolic interaction: the placebo group exhibited significantly greater increases in acetic and propionic acids, while these elevations were attenuated in the CKDB-322 group. Excessive SCFA production has been linked to obesity-related dysbiosis and increased energy harvest in several studies [68,69,70]. The blunted rise in SCFAs observed with CKDB-322 may indicate a more stable and metabolically favorable gut environment. Coupled with the increased relative abundance of *Lactobacillus*, these findings suggest that CKDB-322 may provide functional benefits along the gut–metabolic axis without requiring extensive changes to the gut microbiome.

Preclinical research [40] supports the clinical efficacy demonstrated in this study. In 3T3-L1 adipocytes, CKDB-322 suppressed the adipogenic transcriptional program by downregulating key differentiation regulators, such as PPARγ and C/EBPα, while promoting lipid mobilization. This was evidenced by increased glycerol release and decreased intracellular triglyceride accumulation. In obese mice induced by a high-fat diet, an eight-week administration of CKDB-322 reduced body weight gain, adiposity, and circulating metabolic markers (including glucose, triglycerides, total cholesterol, and leptin) without causing hepatic toxicity. These metabolic improvements were accompanied by a broad inhibition of lipogenesis, as indicated by reduced expression of sterol regulatory element-binding protein-1c (SREBP-1c), acetyl-CoA carboxylase (ACC), and fatty acid synthase (FAS). Additionally, enhanced fatty acid β-oxidation and mitochondrial activity were observed, including upregulation of carnitine palmitoyltransferase 1α (CPT-1α) and peroxisome proliferator-activated receptor α (PPARα). The activation of energy-regulating pathways, such as 5′-AMP-activated protein kinase (AMPK) and PPARγ coactivator-1α (PGC-1α), along with reduced hepatic lipid accumulation and decreased adipocyte hypertrophy, further supported improved lipid handling and metabolic function in adipose tissue.

**Figure 6:**
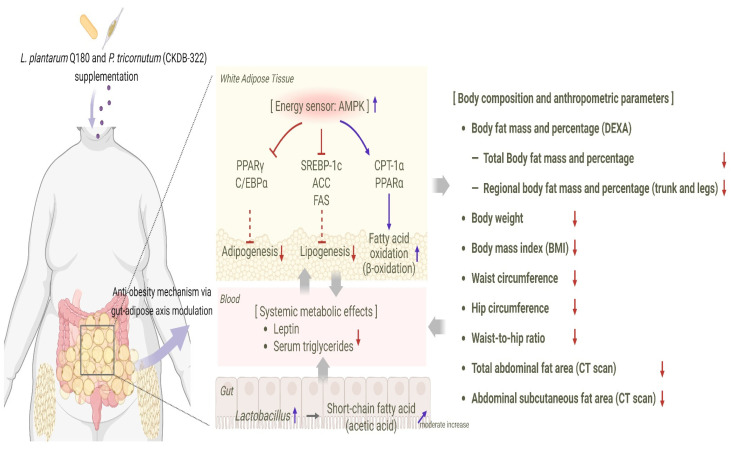
Proposed anti-obesity mechanism of CKDB-322 based on preclinical and clinical findings. Solid arrows indicate direct effects, whereas dashed arrows represent indirect or proposed mechanisms. Blue and red arrows denote increased and decreased expression or production, respectively; arrowheads indicate activation, and blunt-ended lines indicate inhibition. Created in BioRender. Noh, H. (2026) https://BioRender.com/zzvfk7w.

These findings suggest that CKDB-322 affects multiple metabolic pathways by suppressing adipogenesis and lipogenesis, enhancing fatty acid oxidation, and improving adipose tissue endocrine activity through mechanisms involving AMPK activation and the regulation of adipogenic and lipogenic transcription factors. This aligns with the clinical reductions in body fat mass, abdominal adiposity, triglycerides, and leptin observed in this trial, indicating a coordinated improvement in both adipose and systemic metabolic function. Figure 6 presents an integrated summary, demonstrating how CKDB-322 may simultaneously impact adipocyte differentiation, lipid synthesis and breakdown, fatty acid oxidation, and energy-regulatory signaling pathways through modulation of the gut–adipose axis, leading to the favorable clinical outcomes seen in overweight adults.

Additionally, several methodological strengths of this trial bolster confidence in the observed clinical effects. Notably, the study was designed as a randomized, double-blind, placebo-controlled trial, which minimizes bias and enhances causal inference. Primary and secondary outcomes were assessed using objective imaging techniques (DEXA and CT), allowing for precise quantification of overall and regional adiposity. Furthermore, consistent results across both the FAS and PPS populations further validate the robustness of the efficacy findings. The inclusion of exploratory microbiome and SCFA analyses added an extra dimension of mechanistic insight, facilitating a more comprehensive understanding of how CKDB-322 may influence adiposity-related metabolic pathways.

Despite these strengths, several limitations should be acknowledged. It should be noted that reductions in body fat percentage were also observed in the placebo group. This is likely attributable to the standardized dietary counseling and physical activity recommendations provided to all participants as part of the study protocol. Such lifestyle interventions are well known to induce modest reductions in body fat in overweight individuals [71]. Accordingly, the between-group differences observed in this trial are best interpreted as incremental benefits of CKDB-322 beyond standardized lifestyle modification. While the total sample size was sufficient for evaluating the primary endpoint, paired fecal samples for microbiome and SCFA analyses were only available for a subset of participants, which limited the statistical power for these exploratory outcomes. The study population exclusively included overweight adults without significant metabolic comorbidities, potentially restricting the generalizability of the findings to individuals with obesity or established metabolic disorders. Furthermore, the small proportion of male participants resulted in a predominantly female sample. This recruitment pattern aligns with broader epidemiological trends, as women typically exhibit higher body fat percentages and a greater prevalence of obesity than men [72,73], making them more likely to meet adiposity-based eligibility criteria in community-based trials. Nonetheless, this sex imbalance in this study may limit the applicability of the findings to men. To assess whether CKDB-322 is effective for both sexes, future research should include a more balanced representation of male participants. Another limitation is the absence of a post-intervention follow-up period, which restricts our ability to evaluate the durability of the treatment effects. While some reports indicate that the benefits of probiotics may decrease after discontinuation [74], evidence regarding the persistence or decline of these effects is inconsistent. Therefore, longer-term follow-up studies are necessary to determine whether the improvements observed in this trial are maintained after treatment ends. This information is crucial for understanding the practical applicability and long-term management implications of probiotic-based interventions.

Overall, these findings suggest that CKDB-322 is a promising and safe functional ingredient for managing adiposity in overweight adults. Future studies examining long-term effects and including broader populations are essential.

## 5. Conclusions

Twelve weeks of CKDB-322 supplementation resulted in statistically significant reductions in whole-body fat mass, body fat percentage, total abdominal fat area, subcutaneous fat area, and central obesity-related anthropometric indices, including body weight, BMI, waist circumference, hip circumference, and waist-to-hip ratio. In addition, CKDB-322 was associated with significant reductions in serum triglycerides and leptin compared with placebo. These benefits were accompanied by a selective increase in *Lactobacillus* and attenuation of excessive short-chain fatty acid (SCFA) elevations, suggesting that CKDB-322 may influence the gut–adipose metabolic axis. CKDB-322 was safe and well tolerated, with no treatment-related adverse events or clinically relevant laboratory abnormalities. Taken together, these findings suggest that CKDB-322 may serve as a safe and supportive functional ingredient contributing to adiposity management and metabolic health in overweight adults.

## Figures and Tables

**Table 1:** Baseline characteristics of the participants.

	CKDB-322 Group (*n* = 48)	Placebo Group (*n* = 45)	*p*-Value ^(1)^
Sex (M/F)	4/44	6/39	0.5148 ^(2)^
Age (years)	36.46 ± 8.32	35.98 ± 9.33	0.8174
Height (cm)	163.41 ± 6.05	161.51 ± 6.73	0.1213
Weight (kg)	72.56 ± 7.20	70.47 ± 6.63	0.1299
BMI (kg/m^2^)	27.11 ± 1.40	26.98 ± 1.27	0.8175
Waist circumference (cm)	91.08 ± 4.16	90.08 ± 3.81	0.2957
Body fat mass (g)	20,764.52 ± 4429.11	19,228.18 ± 3824.23	0.0776 ^(3)^

Values are presented as mean ± SD or number (%). Between-group comparisons were analyzed by: ^(1)^ Wilcoxon rank-sum test; ^(2)^ Fisher’s exact test; and ^(3)^ Independent *t*-test. BMI, body mass index.

**Table 2:** Changes in dietary intake and physical activity levels before and after 12-week intervention.

	CKDB-322 Group (*n* = 48)			Placebo Group (*n* = 45)			*p*-Value ^(1)^	*p*-Value ^(3)^
	Baseline	12 Week	Change Value	Baseline	12 Week	Change Value		
Energy (kcal)	1240.33 ± 320.46	1130.96 ± 307.15	−109.37 ± 318.58	1229.02 ± 324.88	1156.34 ± 314.67	−72.68 ± 314.99	0.8662	0.5781
Carbohydrates (g)	182.52 ± 45.98	166.28 ± 45.48	−16.24 ± 46.31	176.20 ± 51.93	162.57 ± 52.63	−13.63 ± 52.83	0.5353	0.7999
Lipids (g)	32.31 ± 12.72	29.39 ± 11.44	−2.92 ± 13.90	34.57 ± 12.72	33.93 ± 10.35	−0.64 ± 13.44	0.3787 ^(2)^	0.6474 ^(4)^
Protein (g)	50.80 ± 15.65	46.68 ± 14.42	−4.12 ± 14.28	49.83 ± 14.94	46.77 ± 13.76	−3.06 ± 16.43	0.9479 ^(2)^	0.7393
Fiber (g)	13.27 ± 4.68	11.77 ± 4.45	−1.50 ± 4.57	12.84 ± 4.51	10.96 ± 3.66	−1.89 ± 4.56	0.8566 ^(2)^	0.6850
MET (min/week)	1111.25 ± 1283.25	1576.44 ± 1159.38	465.19 ± 1447.91	1071.92 ± 1283.89	1172.48 ± 1203.17	100.56 ± 1014.60	0.8717 ^(2)^	0.0527 ^(4)^

Values are presented as mean ± SD. Between-group comparisons were analyzed by: ^(1)^ Independent *t*-test at baseline; ^(2)^ Wilcoxon rank-sum test at baseline; ^(3)^ Independent *t*-test for change values; and ^(4)^ Wilcoxon rank-sum test for change values. MET, metabolic equivalent task.

**Table 3:** Changes in body composition measured by DEXA, abdominal fat parameters assessed by CT, and anthropometric parameters before and after 12-week intervention.

	CKDB-322 Group (*n* = 48)				Placebo Group (*n* = 45)				Group Difference	
	Baseline	12 Week	Change Value	*p*-Value ^(1)^	Baseline	12 Week	Change Value	*p*-Value ^(1)^	Mean [95% CI]	*p*-Value ^(3)^
DEXA measurement										
Body fat mass (g)	20,764.52 ± 4429.11	19,205.33 ± 5012.96	−1559.19 ± 1500.58	<0.0001	19,228.18 ± 3824.23	18,564.40 ± 4152.43	−663.78 ± 1523.66	0.0055	−895.4 [−1518.5, −272.3]	0.0053
Body fat percentage (%)	31.17 ± 5.79	29.19 ± 6.29	−1.99 ± 1.68	<0.0001	29.92 ± 5.92	28.83 ± 6.39	−1.09 ± 1.76	0.0001 ^(2)^	−0.89 [−1.60, −0.19]	0.0452 ^(4)^
Lean body mass (g)	46,002.52 ± 6985.42	46,548.42 ± 7101.22	545.90 ± 774.16	<0.0001	45,351.58 ± 6595.54	46,131.44 ± 6686.39	779.87 ± 1051.00	<0.0001	−234.0 [−616.9, 148.9]	0.2276
Abdominal fat CT measurement										
Total abdominal fat area (mm^2^)	32,210.13 ± 6761.02	31,337.15 ± 7318.11	−872.98 ± 3375.92	0.0157 ^(2)^	30,057.13 ± 6033.33	30,555.29 ± 6040.11	498.16 ± 3047.58	0.2788	−1371.1 [−2698.9, 43.4]	0.0086 ^(4)^
Visceral fat area (mm^2^)	8181.29 ± 3442.90	8013.38 ± 3749.14	−167.92 ± 1440.81	0.1251 ^(2)^	7515.11 ± 2954.30	7722.42 ± 3364.80	207.31 ± 1380.54	0.3193	−375.2 [−957.2, 206.8]	0.0976 ^(4)^
Subcutaneous fat area (mm^2^)	24,028.83 ± 5817.77	23,323.77 ± 6181.18	−705.06 ± 2932.64	0.0028 ^(2)^	22,542.02 ± 5816.21	22,832.87 ± 5560.39	290.84 ± 2387.70	0.2964 ^(2)^	−995.9 [−2101.8, 109.9]	0.0196 ^(4)^
Anthropometric parameters										
Weight (kg)	72.56 ± 7.20	70.92 ± 7.69	−1.64 ± 1.76	<0.0001	70.47 ± 6.63	70.61 ± 6.57	0.14 ± 1.53	0.5426	−1.78 [−2.46, −1.10]	<0.0001
BMI (kg/m^2^)	27.11 ± 1.40	26.50 ± 1.72	−0.61 ± 0.66	<0.0001	26.98 ± 1.27	27.04 ± 1.46	0.06 ± 0.60	0.5067	−0.67 [−0.93, −0.41]	<0.0001
Waist circumference (cm)	91.08 ± 4.16	89.11 ± 4.60	−1.97 ± 1.32	<0.0001	90.08 ± 3.81	90.63 ± 3.69	0.55 ± 0.94	0.0003	−2.52 [−2.99, −2.05]	<0.0001
Hip circumference (cm)	101.48 ± 3.94	99.49 ± 4.40	−1.99 ± 1.20	<0.0001	100.03 ± 3.58	100.48 ± 3.38	0.45 ± 0.86	0.0011	−2.43 [−2.86, −2.01]	<0.0001
WHR	0.90 ± 0.03	0.90 ± 0.03	−0.00 ± 0.01	0.0002 ^(2)^	0.90 ± 0.03	0.90 ± 0.04	0.00 ± 0.01	0.0161 ^(2)^	−0.005 [−0.008, −0.002]	0.0004 ^(4)^

Values are presented as mean ± SD. Within-group changes were analyzed by: ^(1)^ Paired *t*-test and ^(2)^ Wilcoxon signed-rank test. Between-group differences in change values were analyzed by: ^(3)^ Independent *t*-test and ^(4)^ Wilcoxon rank-sum test. DEXA, dual-energy X-ray absorptiometry; CT, computed tomography; BMI, body mass index; WHR, Waist-to-Hip ratio; CI, confidence interval.

**Table 4:** Changes in serum lipid profiles, adipokines, and cytokines before and after 12-week intervention.

	CKDB-322 Group (*n* = 48)				Placebo Group (*n* = 45)				*p*-Value ^(3)^
	Baseline	12 Week	Change Value	*p*-Value ^(1)^	Baseline	12 Week	Change Value	*p*-Value ^(1)^	
Lipid profiles									
Total cholesterol (mg/dL)	195.38 ± 35.00	199.69 ± 32.80	4.31 ± 28.14	0.2937	203.80 ± 35.59	208.91 ± 38.23	5.11 ± 25.16	0.1799	0.8859
Triglyceride (mg/dL)	123.85 ± 73.59	106.38 ± 44.24	−17.48 ± 61.32	0.0884 ^(2)^	122.91 ± 88.12	135.24 ± 102.76	12.33 ± 53.06	0.1858 ^(2)^	0.0211 ^(4)^
HDL-C (mg/dL)	53.17 ± 9.28	55.40 ± 9.14	2.23 ± 7.28	0.0063 ^(2)^	56.87 ± 13.85	58.36 ± 13.52	1.49 ± 6.34	0.1222	0.4056 ^(4)^
LDL-C (mg/dL)	125.31 ± 34.83	129.79 ± 28.35	4.48 ± 25.93	0.2374	129.31 ± 31.40	129.49 ± 30.16	0.18 ± 23.05	0.959	0.4012
Adipokines, cytokines									
Adiponectin (ng/mL)	8571.98 ± 4406.46	8379.48 ± 4819.18	−192.50 ± 2039.55	0.3946 ^(2)^	9947.44 ± 5115.76	9416.67 ± 4971.15	−530.78 ± 2238.59	0.0425 ^(2)^	0.4217 ^(4)^
Leptin (ng/mL)	28.78 ± 14.44	24.64 ± 14.74	−4.14 ± 10.18	0.0071	27.91 ± 15.28	31.56 ± 15.79	3.65 ± 11.02	0.0317	0.0006
TNF-α (pg/mL)	0.53 ± 0.14	0.53 ± 0.17	0.00 ± 0.15	0.7922 ^(2)^	0.52 ± 0.12	0.53 ± 0.15	0.00 ± 0.12	0.8662	0.9755 ^(4)^
IL-1β (pg/mL)	0.08 ± 0.09	0.07 ± 0.11	−0.01 ± 0.05	0.1924 ^(2)^	0.07 ± 0.05	0.06 ± 0.04	−0.01 ± 0.06	0.1583 ^(2)^	0.8235 ^(4)^
IL-6 (pg/mL)	1.68 ± 0.98	1.72 ± 1.00	0.04 ± 0.84	0.7192 ^(2)^	1.61 ± 1.32	1.71 ± 1.42	0.10 ± 0.93	0.7942 ^(2)^	0.9295 ^(4)^

Values are presented as mean ± SD. Within-group changes were analyzed by: ^(1)^ Paired *t*-test and ^(2)^ Wilcoxon signed-rank test. Between-group differences in change values were analyzed by: ^(3)^ Independent *t*-test and ^(4)^ Wilcoxon rank-sum test.

**Table 5:** Changes in short-chain fatty acids before and after 12-week intervention.

	CKDB-322 Group (*n* = 32)				Placebo Group (*n* = 28)				*p*-Value ^(3)^
	Baseline	12 Week	Change Value	*p*-Value ^(1)^	Baseline	12 Week	Change Value	*p*-Value ^(1)^	
Acetic Acid (µmol/g)	31.71 ± 22.61	45.15 ± 26.21	13.44 ± 37.32	0.0433	31.72 ± 22.38	60.41 ± 26.46	28.69 ± 28.85	<0.0001 ^(2)^	0.0843
Propionic Acid (µmol/g)	27.14 ± 26.24	27.52 ± 17.71	0.381 ± 33.57	0.6117	23.26 ± 18.30	36.25 ± 21.39	13.00 ± 25.13	0.0048	0.0765
Butyric Acid (µmol/g)	41.09 ± 27.96	45.05 ± 22.52	3.962 ± 38.69	0.2781	36.27 ± 22.73	45.38 ± 19.41	9.111 ± 24.53	0.1375	0.6643

Values are presented as mean ± SD. Within-group changes were analyzed by: ^(1)^ Wilcoxon signed-rank test and ^(2)^ Paired *t*-test. Between-group differences in change values were analyzed by: ^(3)^ Wilcoxon rank-sum test.

## Data Availability

The datasets generated and analyzed during the current study are available from the corresponding author on reasonable request. The data are not publicly available due to privacy and ethical restrictions.

## Supplementary Materials

The following supporting information can be downloaded at https://www.mdpi.com/article/10.3390/nu18020250/s1, Table S1: Changes in efficacy outcomes before and after 12-week intervention (PPS).

## References

[1] Camacho S., Ruppel A. (2017). Is the calorie concept a real solution to the obesity epidemic?. Glob. Health Action.

[2] Hruby A., Hu F.B. (2015). The epidemiology of obesity: A big picture. Pharmacoeconomics.

[3] Simo L.P., Agbor V.N., Temgoua F.Z., Fozeu L.C.F., Bonghaseh D.T., Mbonda A.G.N., Yurika R., Dotse-Gborgbortsi W., Mbanya D. (2021). Prevalence and factors associated with overweight and obesity in selected health areas in a rural health district in Cameroon: A cross-sectional analysis. BMC Public Health.

[4] Jayedi A., Rashidy-Pour A., Khorshidi M., Shab-Bidar S. (2018). Body mass index, abdominal adiposity, weight gain and risk of developing hypertension: A systematic review and dose–response meta-analysis of more than 2.3 million participants. Obes. Rev..

[5] Klop B., Elte J.W.F., Castro Cabezas M. (2013). Dyslipidemia in obesity: Mechanisms and potential targets. Nutrients.

[6] Caspard H., Jabbour S., Hammar N., Fenici P., Sheehan J.J., Kosiborod M. (2018). Recent trends in the prevalence of type 2 diabetes and the association with abdominal obesity lead to growing health disparities in the USA: An analysis of the NHANES surveys from 1999 to 2014. Diabetes Obes. Metab..

[7] Kotsis V., Tsioufis K., Antza C., Seravalle G., Coca A., Sierra C., Lurbe E., Stabouli S., Jelakovic B., Redon J. (2018). Obesity and cardiovascular risk: A call for action from the European Society of Hypertension Working Group of Obesity, Diabetes and the High-risk Patient and European Association for the Study of Obesity: Part B: Obesity-induced cardiovascular disease, early prevention strategies and future research directions. J. Hypertens..

[8] Van Raemdonck K., Umar S., Szekanecz Z., Zomorrodi R.K., Shahrara S. (2018). Impact of obesity on autoimmune arthritis and its cardiovascular complications. Autoimmun. Rev..

[9] Lovren F., Teoh H., Verma S. (2015). Obesity and atherosclerosis: Mechanistic insights. Can. J. Cardiol..

[10] Ottaiano A., De Divitiis C., Capozzi M., Avallone A., Pisano C., Pignata S., Tafuto S. (2018). Obesity and cancer: Biological links and treatment implications. Curr. Cancer Drug Targets.

[11] Kim M.K., Kim C.S. (2018). Recent advances in anti-obesity agents. Korean J. Med..

[12] Kim T.N. (2020). Barriers to obesity management: Patient and physician factors. J. Obes. Metab. Syndr..

[13] Wadden T.A., Tronieri J.S., Butryn M.L. (2020). Lifestyle modification approaches for the treatment of obesity in adults. Am. Psychol..

[14] Müller T.D., Blüher M., Tschöp M.H., DiMarchi R.D. (2022). Anti-obesity drug discovery: Advances and challenges. Nat. Rev. Drug Discov..

[15] Alsuhibani A., Alrasheed M., Gari M., Hincapie A.L., Guo J.J. (2022). Descriptive analysis of reported adverse events associated with anti-obesity medications using FDA Adverse Event Reporting System (FAERS) databases 2013–2020. Int. J. Clin. Pharm..

[16] Krentz A., Fujioka K., Hompesch M. (2016). Evolution of pharmacological obesity treatments: Focus on adverse side-effect profiles. Diabetes Obes. Metab..

[17] Hill C., Guarner F., Reid G., Gibson G.R., Merenstein D.J., Pot B., Morelli L., Canani R.B., Flint H.J., Salminen S. (2014). Expert consensus document: The International Scientific Association for Probiotics and Prebiotics consensus statement on the scope and appropriate use of the term probiotic. Nat. Rev. Gastroenterol. Hepatol..

[18] Wiciński M., Gębalski J., Gołębiewski J., Malinowski B. (2020). Probiotics for the treatment of overweight and obesity in humans—A review of clinical trials. Microorganisms.

[19] Turnbaugh P.J., Ley R.E., Mahowald M.A., Magrini V., Mardis E.R., Gordon J.I. (2006). An obesity-associated gut microbiome with increased capacity for energy harvest. Nature.

[20] Cani P.D., Delzenne N.M. (2009). The role of the gut microbiota in energy metabolism and metabolic disease. Curr. Pharm. Des..

[21] Esteve E., Ricart W., Fernandez-Real J.-M. (2011). Gut microbiota interactions with obesity, insulin resistance and type 2 diabetes: Did gut microbiote co-evolve with insulin resistance?. Curr. Opin. Clin. Nutr. Metab. Care.

[22] Cox T.O., Lundgren P., Nath K., Thaiss C.A. (2022). Metabolic control by the microbiome. Genome Med..

[23] Mekkes M., Weenen T., Brummer R.J., Claassen E. (2014). The development of probiotic treatment in obesity: A review. Benef. Microbes.

[24] Kleerebezem M., Hols P., Bernard E., Rolain T., Zhou M., Siezen R.J., Bron P.A. (2010). The extracellular biology of the lactobacilli. FEMS Microbiol. Rev..

[25] Zheng J., Wittouck S., Salvetti E., Franz C.M., Harris H.M., Mattarelli P., O’toole P.W., Pot B., Vandamme P., Walter J. (2020). A taxonomic note on the genus *Lactobacillus*: Description of 23 novel genera, emended description of the genus *Lactobacillus* Beijerinck 1901, and union of *Lactobacillaceae* and *Leuconostocaceae*. Int. J. Syst. Evol. Microbiol..

[26] Salminen S., von Wright A., Morelli L., Marteau P., Brassart D., de Vos W.M., Fondén R., Saxelin M., Collins K., Mogensen G. (1998). Demonstration of safety of probiotics—A review. Int. J. Food Microbiol..

[27] Choi M.J., Yu H., Kim J.I., Seo H., Kim J.G., Kim S.-K., Lee H.S., Cheon H.G. (2023). Anti-obesity effects of *Lactiplantibacillus* plantarum SKO-001 in high-fat diet-induced obese mice. Eur. J. Nutr..

[28] Jang A.-R., Jung D.-H., Lee T.-S., Kim J.-K., Lee Y.-B., Lee J.-Y., Kim S.-Y., Yoo Y.-C., Ahn J.-H., Hong E.-H. (2024). *Lactobacillus plantarum* NCHBL-004 modulates high-fat diet–induced weight gain and enhances GLP-1 production for blood glucose regulation. Nutrition.

[29] Zhao L., Shen Y., Wang Y., Wang L., Zhang L., Zhao Z., Li S. (2022). *Lactobacillus plantarum* S9 alleviates lipid profile, insulin resistance, and inflammation in high-fat diet-induced metabolic syndrome rats. Sci. Rep..

[30] Li C.-P., Chen C.-C., Hsiao Y., Kao C.-H., Chen C.-C., Yang H.-J., Tsai R.-Y. (2024). The role of *Lactobacillus plantarum* in reducing obesity and inflammation: A meta-analysis. Int. J. Mol. Sci..

[31] Park Y.E., Kim M.S., Shim K.W., Kim Y.-I., Chu J., Kim B.-K., Choi I.S., Kim J.Y. (2020). Effects of Lactobacillus plantarum Q180 on postprandial lipid levels and intestinal environment: A double-blind, randomized, placebo-controlled, parallel trial. Nutrients.

[32] Park S.-Y., Cho S.-A., Kim S.-H., Lim S.-D. (2014). Physiological characteristics and anti-obesity effect of *Lactobacillus plantarum* Q180 isolated from feces. Korean J. Food Sci. Anim. Resour..

[33] Park S.-Y., Cho S.-A., Lim S.-D. (2014). Application of response surface methodology (RSM) for optimization of anti-obesity effect in fermented milk by *Lactobacillus plantarum* Q180. Korean J. Food Sci. Anim. Resour..

[34] Park S.-Y., Seong K.-S., Lim S.-D. (2016). Anti-obesity effect of yogurt fermented by *Lactobacillus plantarum* Q180 in diet-induced obese rats. Korean J. Food Sci. Anim. Resour..

[35] Park S.-Y., Kim S., Lim S.-D. (2018). The inhibitory effect of *L. plantarum* Q180 on adipocyte differentiation in 3T3-L1 and reduction of adipocyte size in mice fed high-fat diet. Korean J. Food Sci. Anim. Resour..

[36] Derwenskus F., Schäfer B., Müller J., Frick K., Gille A., Briviba K., Schmid-Staiger U., Hirth T. (2020). Coproduction of EPA and fucoxanthin with *P. tricornutum*—A promising approach for up-and downstream processing. Chem. Ing. Tech..

[37] Gammone M.A., D’Orazio N. (2015). Anti-obesity activity of the marine carotenoid fucoxanthin. Mar. Drugs.

[38] Kim J.H., Kim S.M., Cha K.H., Mok I.-K., Koo S.Y., Pan C.-H., Lee J.K. (2016). Evaluation of the anti-obesity effect of the microalga Phaeodactylum tricornutum. Appl. Biol. Chem..

[39] Koo S.Y., Hwang J.-H., Yang S.-H., Um J.-I., Hong K.W., Kang K., Pan C.-H., Hwang K.T., Kim S.M. (2019). Anti-obesity effect of standardized extract of microalga *Phaeodactylum tricornutum* containing fucoxanthin. Mar. Drugs.

[40] Noh H.-J., Eom J.-I., Park S.-J., Shin C.H., Kim S.-M., Pan C.-H., Lee J.K. (2025). Synergistic Anti-Obesity Effects of *Lactiplantibacillus plantarum* Q180 and *Phaeodactylum tricornutum* (CKDB-322) in High-Fat-Diet-Induced Obese Mice. Int. J. Mol. Sci..

[41] WHO Consultation (2000). Obesity: Preventing and Managing the Global Epidemic.

[42] Kadooka Y., Sato M., Ogawa A., Miyoshi M., Uenishi H., Ogawa H., Ikuyama K., Kagoshima M., Tsuchida T. (2013). Effect of *Lactobacillus gasseri* SBT2055 in fermented milk on abdominal adiposity in adults in a randomised controlled trial. Br. J. Nutr..

[43] Jeon S.M., Kim H.J., Woo M.N., Lee M.K., Shin Y.C., Park Y.B., Choi M.S. (2010). Fucoxanthin-rich seaweed extract suppresses body weight gain and improves lipid metabolism in high-fat-fed C57BL/6J mice. Biotechnol. J..

[44] Woo M.-N., Jeon S.-M., Kim H.-J., Lee M.-K., Shin S.-K., Shin Y.C., Park Y.-B., Choi M.-S. (2010). Fucoxanthin supplementation improves plasma and hepatic lipid metabolism and blood glucose concentration in high-fat fed C57BL/6N mice. Chem.-Biol. Interact..

[45] Beppu F., Hosokawa M., Niwano Y., Miyashita K. (2012). Effects of dietary fucoxanthin on cholesterol metabolism in diabetic/obese KK-A y mice. Lipids Health Dis..

[46] Maeda H., Hosokawa M., Sashima T., Murakami-Funayama K., Miyashita K. (2009). Anti-obesity and anti-diabetic effects of fucoxanthin on diet-induced obesity conditions in a murine model. Mol. Med. Rep..

[47] Woo M.N., Jeon S.M., Shin Y.C., Lee M.K., Kang M.A., Choi M.S. (2009). Anti-obese property of fucoxanthin is partly mediated by altering lipid-regulating enzymes and uncoupling proteins of visceral adipose tissue in mice. Mol. Nutr. Food Res..

[48] Dickerson B., Maury J., Jenkins V., Nottingham K., Xing D., Gonzalez D.E., Leonard M., Kendra J., Ko J., Yoo C. (2024). Effects of supplementation with microalgae extract from *Phaeodactylum tricornutum* (Mi136) to support benefits from a weight management intervention in overweight women. Nutrients.

[49] Haarbo J., Gotfredsen A., Hassager C., Christiansen C. (1991). Validation of body composition by dual energy X-ray absorptiometry (DEXA). Clin. Physiol..

[50] Shepherd J.A., Ng B.K., Sommer M.J., Heymsfield S.B. (2017). Body composition by DXA. Bone.

[51] Lee S.-B., Yoo B., Baeg C., Yun J., Ryu D.-w., Kim G., Kim S., Shin H., Lee J.H. (2025). A 12-Week, Randomized, Double-Blind, Placebo-Controlled Study to Evaluate the Efficacy and Safety of Lactobacillus plantarum LMT1-48 on Body Fat Loss. Nutrients.

[52] Shin S.M., Park J.-S., Kim S.B., Cho Y.H., Seo H., Lee H.S. (2024). A 12-week, single-centre, randomised, double-blind, placebo-controlled, parallel-design clinical trial for the evaluation of the efficacy and safety of *Lactiplantibacillus plantarum* SKO-001 in reducing body fat. Nutrients.

[53] Koutnikova H., Genser B., Monteiro-Sepulveda M., Faurie J.-M., Rizkalla S., Schrezenmeir J., Clément K. (2019). Impact of bacterial probiotics on obesity, diabetes and non-alcoholic fatty liver disease related variables: A systematic review and meta-analysis of randomised controlled trials. BMJ Open.

[54] Magkos F., Fraterrigo G., Yoshino J., Luecking C., Kirbach K., Kelly S.C., de las Fuentes L., He S., Okunade A.L., Patterson B.W. (2016). Effects of Moderate and Subsequent Progressive Weight Loss on Metabolic Function and Adipose Tissue Biology in Humans with Obesity. Cell Metab..

[55] Kuk J.L., Katzmarzyk P.T., Nichaman M.Z., Church T.S., Blair S.N., Ross R. (2006). Visceral fat is an independent predictor of all-cause mortality in men. Obesity.

[56] Hendler R.G., Welle S.L., Statt M.C., Barnard R., Amatruda J.M. (1995). The effects of weight reduction to ideal body weight on body fat distribution. Metabolism.

[57] Stiegler P., Cunliffe A. (2006). The role of diet and exercise for the maintenance of fat-free mass and resting metabolic rate during weight loss. Sports Med..

[58] Nelson K.M., Weinsier R.L., Long C.L., Schutz Y. (1992). Prediction of resting energy expenditure from fat-free mass and fat mass. Am. J. Clin. Nutr..

[59] Kvist H., Chowdhury B., Grangård U., Tylen U., Sjöström L. (1988). Total and visceral adipose-tissue volumes derived from measurements with computed tomography in adult men and women: Predictive equations. Am. J. Clin. Nutr..

[60] Fox C.S., Massaro J.M., Hoffmann U., Pou K.M., Maurovich-Horvat P., Liu C.-Y., Vasan R.S., Murabito J.M., Meigs J.B., Cupples L.A. (2007). Abdominal visceral and subcutaneous adipose tissue compartments: Association with metabolic risk factors in the Framingham Heart Study. Circulation.

[61] Booth A., Magnuson A., Fouts J., Wei Y., Wang D., Pagliassotti M., Foster M. (2018). Subcutaneous adipose tissue accumulation protects systemic glucose tolerance and muscle metabolism. Adipocyte.

[62] Perna S., Ilyas Z., Giacosa A., Gasparri C., Peroni G., Faliva M.A., Rigon C., Naso M., Riva A., Petrangolini G. (2021). Is probiotic supplementation useful for the management of body weight and other anthropometric measures in adults affected by overweight and obesity with metabolic related diseases? A systematic review and meta-analysis. Nutrients.

[63] Pagliai G., Coman M.M., Baldi S., Dinu M., Nannini G., Russo E., Curini L., Colombini B., Lotti S., Pallecchi M. (2023). Effects of the probiotic *Lactiplantibacillus plantarum* IMC 510^®^ on body composition, biochemical parameters, gut microbiota composition and function, and clinical symptoms of overweight/obese subjects. Front. Nutr..

[64] Mulligan A.A., Lentjes M.A., Luben R.N., Wareham N.J., Khaw K.-T. (2019). Changes in waist circumference and risk of all-cause and CVD mortality: Results from the European Prospective Investigation into Cancer in Norfolk (EPIC-Norfolk) cohort study. BMC Cardiovasc. Disord..

[65] Forny-Germano L., De Felice F.G., Vieira M.N.d.N. (2019). The role of leptin and adiponectin in obesity-associated cognitive decline and Alzheimer’s disease. Front. Neurosci..

[66] Cambuli V.M., Musiu M.C., Incani M., Paderi M., Serpe R., Marras V., Cossu E., Cavallo M.G., Mariotti S., Loche S. (2008). Assessment of adiponectin and leptin as biomarkers of positive metabolic outcomes after lifestyle intervention in overweight and obese children. J. Clin. Endocrinol. Metab..

[67] Zhao S., Zhu Y., Schultz R.D., Li N., He Z., Zhang Z., Caron A., Zhu Q., Sun K., Xiong W. (2019). Partial leptin reduction as an insulin sensitization and weight loss strategy. Cell Metab..

[68] Murugesan S., Nirmalkar K., Hoyo-Vadillo C., García-Espitia M., Ramírez-Sánchez D., García-Mena J. (2018). Gut microbiome production of short-chain fatty acids and obesity in children. Eur. J. Clin. Microbiol. Infect. Dis..

[69] Cani P.D., Van Hul M., Lefort C., Depommier C., Rastelli M., Everard A. (2019). Microbial regulation of organismal energy homeostasis. Nat. Metab..

[70] Breton J., Galmiche M., Déchelotte P. (2022). Dysbiotic gut bacteria in obesity: An overview of the metabolic mechanisms and therapeutic perspectives of next-generation probiotics. Microorganisms.

[71] Oraphruek P., Chusak C., Ngamukote S., Sawaswong V., Chanchaem P., Payungporn S., Suantawee T., Adisakwattana S. (2023). Effect of a multispecies synbiotic supplementation on body composition, antioxidant status, and gut microbiomes in overweight and obese subjects: A randomized, double-blind, placebo-controlled study. Nutrients.

[72] Kanter R., Caballero B. (2012). Global gender disparities in obesity: A review. Adv. Nutr..

[73] Park H.S., Yun Y.S., Park J.Y., Kim Y.S., Choi J.M. (2003). Obesity, abdominal obesity, and clustering of cardio-vascular risk factors in South Korea. Asia Pac. J. Clin. Nutr..

[74] Zeilstra D., Younes J.A., Brummer R.J., Kleerebezem M. (2018). Perspective: Fundamental limitations of the randomized controlled trial method in nutritional research: The example of probiotics. Adv. Nutr..

