# Supplementary material for: Efficacy and Safety of CKDB-322, a Combination of Lactiplantibacillus plantarum Q180 and Phaeodactylum tricornutum, for Reducing Body Fat and Abdominal Adiposity in Overweight Adults
Source: Nutrients. 2026 Jan 13;18(2):250. doi: 10.3390/nu18020250 (PMC12845410; doi:10.3390/nu18020250)
Supplement: Supplementary file 1 [file nutrients-18-00250-s001.zip › nutrients-4088400-supplementary.pdf]

Table S1: Changes in efficacy outcomes before and after 12-week intervention (**PPS**)

	**CKDB-322 group (n=47)**				**Placebo group (n=44)**				***p*-value[3)]**
	**Baseline**	**12 week**	**Change Value**	***p*-value[1)]**	**Baseline**	**12 week**	**Change Value**	***p*-value[1)]**	
**DEXA measurement**									
Body fat mass (g)	20,759.70±4,476.86	19,145.32±5,049.69	-1,614.38±1,466.72	<.0001	19,217.82±3,867.80	18,629.18±4,177.37	-588.64±1,454.49	0.0103	0.0012
Body fat percentage (%)	31.10±5.83	29.06±6.30	-2.04±1.66	<.0001	29.89±5.99	28.87±6.46	-1.02±1.71	0.0003[2)]	0.0215[4)]
Lean body mass (g)	46,138.23±6,996.68	46,670.87±7,126.58	532.64±777.00	<.0001	45,397.75±6,664.43	46,220.61±6,736.57	822.86±1,022.33	<.0001	0.1294
**Abdominal fat CT measurement**									
Total abdominal fat area (mm^2)	32,324.26±6,787.22	31,320.11±7,396.27	-1,004.15±3,286.45	0.0061[2)]	29,997.50±6,089.66	30,488.73±6,093.22	491.23±3,082.46	0.2964	0.0057[4)]
Visceral fat area (mm^2)	8,202.13±3,477.06	7,991.98±3,786.71	-210.15±1,426.04	0.0733[2)]	7,546.11±2,981.04	7,769.02±3,388.98	222.91±1,392.48	0.2942	0.0614[4)]
Subcutaneous fat area (mm^2)	24,122.13±5,844.26	23,328.13±6,247.93	-794.00±2,898.17	0.0008[2)]	22,451.39±5,851.22	22,719.70±5,572.01	268.32±2,410.46	0.4643	0.0156[4)]
**Anthropometric parameters**									
Weight (kg)	72.68±7.22	70.96±7.77	-1.72±1.67	<.0001	70.50±6.70	70.75±6.58	0.25±1.37	0.2421	<.0001
BMI (kg/m^2)	27.13±1.41	26.48±1.73	-0.64±0.63	<.0001	27.02±1.26	27.12±1.38	0.10±0.54	0.2296	<.0001
Waist circumference (cm)	91.04±4.20	89.02±4.61	-2.02±1.28	<.0001	90.05±3.84	90.66±3.72	0.62±0.85	<.0001	<.0001
Hip circumference (cm)	101.52±3.98	99.48±4.44	-2.04±1.15	<.0001	100.03±3.62	100.53±3.40	0.51±0.78	<.0001	<.0001
WHR	0.90±0.03	0.89±0.03	-0.00±0.01	0.0002[2)]	0.90±0.03	0.90±0.04	0.00±0.01	0.0161[2)]	0.0004[4)]
Lipid profiles									
Total cholesterol (mg/dL)	195.17±35.35	199.11±32.91	3.94±28.32	0.3456	203.80±36.00	207.77±37.89	3.98±24.26	0.2828	0.9941
Triglyceride (mg/dL)	124.19±74.35	104.64±43.03	-19.55±60.25	0.0470[2)]	123.32±89.10	136.82±103.40	13.50±53.09	0.1292[2)]	0.0086[4)]
HDL-C (mg/dL)	53.13±9.38	55.30±9.21	2.17±7.35	0.0093[2)]	56.75±13.98	58.09±13.56	1.34±6.33	0.1673	0.3561[4)]
LDL-C (mg/dL)	125.11±35.18	129.81±28.66	4.70±26.16	0.2241	129.18±31.75	128.39±29.58	-0.80±22.36	0.8146	0.2857
Adipokines, cytokines									
Adiponectin (ng/mL)	8,543.83±4,449.73	8,401.38±4,868.86	-142.45±2,031.58	0.4974[2)]	9,930.45±5,173.62	9,395.23±5,026.51	-535.23±2,264.27	0.0516[2)]	0.3548[4)]
Leptin (ng/mL)	28.70±14.58	24.50±14.87	-4.20±10.28	0.0074	27.99±15.45	31.91±15.79	3.92±10.99	0.0226	0.0005
TNF-α (pg/mL)	0.53±0.14	0.53±0.17	0.00±0.15	0.6769[2)]	0.53±0.12	0.53±0.15	-0.00±0.12	0.9306	0.8055[4)]
IL-1β (pg/mL)	0.08±0.09	0.07±0.11	-0.01±0.06	0.1620[2)]	0.08±0.05	0.06±0.04	-0.01±0.06	0.1583[2)]	0.8926[4)]
IL-6 (pg/mL)	1.68±0.99	1.72±1.01	0.04±0.84	0.6592[2)]	1.63±1.33	1.73±1.42	0.11±0.94	0.7091[2)]	0.9335[4)]

Values are presented as mean ± SD. Within-group changes were analyzed by: [1)]Paired t-test and [2)]Wilcoxon signed-rank test. Between-group differences in change values were analyzed by: [3)]Independent t-test and [4)]Wilcoxon rank-sum test. PPS, per-protocol set; DEXA, dual-energy X-ray absorptiometry; CT, computed tomography; BMI, body mass index; WHR, Waist-to-Hip ratio
